# Adverse childhood experiences and personality traits associate with excessive fatigue in Norwegian nurses

**DOI:** 10.3389/fpsyg.2026.1771618

**Published:** 2026-03-10

**Authors:** Stand Hiestand, Ståle Pallesen, Ingeborg Forthun, Siri Waage, Truls Østbye, Øystein Vedaa, Bjørn Bjorvatn

**Affiliations:** 1 Department of Global Public Health and Primary Care, University of Bergen, Bergen, Norway; 2 Norwegian Competence Center for Sleep Disorders, Haukeland University Hospital, Bergen, Norway; 3 Department of Psychosocial Science, University of Bergen, Bergen, Norway; 4 Department of Disease Burden, Norwegian Institute of Public Health, Bergen, Norway; 5 Department of Family Medicine and Community Health, Duke University, Durham, NC, United States; 6 Department of Health Promotion, Norwegian Institute of Public Health, Bergen, Norway; 7 Solli District Psychiatric Centre (DPS), Bergen, Norway

**Keywords:** early life trauma, big five, workaholism neuroticism, circadian type, circadian preference, fatigue

## Abstract

**Introduction:**

Nurse fatigue may cause medical errors, absence and turnover. Prior research has largely emphasized modifiable factors like work schedules. This focus may overlook non‑modifiable individual factors that also meaningfully contribute to fatigue risk. This study therefore aimed to investigate the relationship between potentially non-modifiable factors and excessive fatigue in nurses.

**Methods:**

This longitudinal cohort study investigated adverse childhood experiences and personality traits in relation to excessive fatigue in nurses. Adverse childhood experiences were assessed with four questions previously used in the Norwegian context. Personality traits included the Big Five traits (Mini-IPIP), morningness-eveningness (Horne and Östberg Morningness-Eveningness Questionnaire reduced scale), circadian type (flexibility and languidity, Circadian Type Inventory), and workaholism (Bergen Work Addiction Scale). Questionnaire data was collected at various time points from an ongoing cohort study known as the Survey of Shift work, Sleep, and Health (REK VEST, no. 088.88). Fatigue was assessed using the Chalder Fatigue Questionnaire and excessive fatigue was considered as scores of ≥4. The study sample included 741 non-pregnant Norwegian nurses. Logistic regression analyses were used to investigate associations between adverse childhood experiences, personality traits, and excessive fatigue.

**Results:**

Nurses who lacked a trusted adult in childhood (adjusted odds ratios (aOR) = 4.68, 95% CI = 1.97–11.11), reported bad memories (aOR = 4.94, CI = 2.70–9.03), or perceived their childhood as difficult (aOR = 4.53, CI = 2.40–8.57) had >4x the odds of excessive fatigue. High neuroticism (aOR = 2.37, CI = 1.56–3.59), low conscientiousness (aOR = 2.02, CI = 1.30–3.12), and high languidity (aOR = 5.01 CI = 2.98–8.39) increased odds of excessive fatigue. Morning types had lower (aOR = 0.65 CI = 0.45–0.93), while evening types had higher odds (aOR = 1.55 CI = 1.02–2.34) of excessive fatigue compared to intermediate types. Lastly, workaholism increased odds of excessive fatigue (aOR = 2.70 CI = 1.21–6.04).

**Discussion:**

In past literature, nurse fatigue has been studied in the context of pain, (shift)work, mental-health and other modifiable factors. This study indicates factors that are less modifiable and potentially difficult to address within the working environment, including adverse childhood experiences and personality traits, may nonetheless play important roles in excessive fatigue in nurses.

## 1 Introduction

Fatigue can be described as an awareness of diminished capacity for activities (Aaronson et al., 1999). It can range from acute—normative daily fatigue—to pathological (Techera et al., 2016; Jason et al., 2010). Fatigue can encompass physical and/or mental components, physical (muscular) fatigue decreases muscular force capability, whereas mental fatigue is a perception of tiredness or low energy (Pallesen and Bjorvatn, 2017).

Occupational fatigue comprises physical and mental exhaustion (Pi et al., 2025). Distinguishing between these dimensions may be challenging in real-world settings. Therefore, this study utilized a global fatigue score from the Chalder Fatigue Questionnaire (Chalder et al., 1993) that includes both dimensions. In this study, excessive fatigue refers to fatigue exceeding normative acute levels, operationalized using the case definition proposed by Chalder et al. (1993; scoring ≥4 bimodally on the Chalder Fatigue Questionnaire).

Fatigue affects about 20% of the general population (Yoon et al., 2023) and can be related to disease, mental health, workplace factors (Rose et al., 2017) and sleep problems (Lichstein et al., 1997). Nurses are notably exposed to factors that may increase fatigue including shift work (Rose et al., 2017) and poor sleep (Sun et al., 2019). Fatigue in nurses may lead to errors, absences and resignations (Cho and Steege, 2021). In the context of “a global crisis” of nursing shortages (Oulton, 2006) fatigue’s associations with high rates of absence (Roelen et al., 2013) and turnover (Søbstad et al., 2021) underlines its importance in a nursing population. In 2018, 35.4% of nurses participating in the Survey of Shift work, Sleep, and Health (SUSSH) reported excessive fatigue (Hiestand et al., 2023). Another study from this cohort found associations between excessive fatigue and lifestyle, sleep and psychosocial work factors, but not shift work schedules (Hiestand et al., 2024).

Adverse childhood experiences are linked to negative physical and psychological outcomes (Petruccelli et al., 2019) and higher risk of chronic fatigue syndrome (Heim et al., 2006). Additionally, there is a strong connection between having multiple adverse childhood experiences concurrently and risk of a number of health conditions (Hughes et al., 2017). While research on adverse childhood experiences in nurses is limited, positive correlations have been found between adverse childhood experiences and burnout (Williamson et al., 2025; Xu et al., 2025), sleep quality (Jung and Oh, 2020), anxiety, depression, and stress (Gürsoy and Mechmet, 2023) in nurses or nursing students. The relationship between adverse childhood experiences and excessive fatigue in nurses remains to be explored.

Personality traits have been investigated in relation to fatigue in some settings (Saksvik-Lehouillier et al., 2012; Stephan et al., 2022). Neuroticism (De Gucht et al., 2003; Stephan et al., 2022; Sørengaard et al., 2019; West et al., 2007) and languidity (propensity toward sleepiness/tiredness from sleep loss; Saksvik-Lehouillier et al., 2012; West et al., 2007) have been reported to be positively associated with fatigue, while flexibility (the ability to sleep and work at odd times) has been reported to be negatively associated (Saksvik-Lehouillier et al., 2013). While inconsistent, inverse associations between extraversion and conscientiousness with fatigue have been found (Michielsen et al., 2007; Stephan et al., 2022). In one study, extraverted and conscientious behaviors were associated with lower levels of fatigue while engaging in the given behavior, but higher levels of fatigue 3 h afterwards (Leikas and Ilmarinen, 2017). Additionally, extroverted nurses from Iran had lower (more favorable) mean scores than introverted nurses on “sleep and fatigue” as assessed using the Standard Shift Work Index (Farzianpour et al., 2015), while extraversion was not associated with chronic fatigue at either 6 or 12 months in a cohort of newly graduated Australian nurses (West et al., 2007).

Circadian preference may also play a role, but again results are mixed. Evening types reported more severe fatigue than morning or intermediate types among Turkish nurses (Karahan et al., 2020). However, no significant relationship was found between morningness-eveningness and chronic fatigue among the newly graduated Australian cohort of nurses 6 to 12 months after beginning work (West et al., 2007). A Polish study on nurses that examined the interactive effect of personality traits found that interactions between neuroticism and languidity correlated positively while interactions between flexibility and extroversion correlated negatively with chronic fatigue (Iskra-Golec et al., 1995). Additionally, increased evening fatigue has been observed on days with higher workaholic behavior in American workers (Clark et al., 2021).

There are notable gaps within the academic literature on nurses. While studies among healthcare workers have investigated the relationships of adverse childhood experiences and personality traits with burnout (Barbosa et al., 2024; Divinakumar et al., 2019; Liang et al., 2025; Lu et al., 2022; Milne et al., 2024; Nonnis et al., 2018; Pelin et al., 2023; Trockel et al., 2023; Williamson et al., 2025; Xu et al., 2025) and compassion fatigue (Bouchard and Rainbow, 2021; Çiçek Korkmaz and Gökoğlan, 2024; Gallardo and Rohde, 2018; Williamson et al., 2025; Zhao and Xie, 2025), fatigue as a distinct outcome has received comparatively little attention.

Existing nursing studies that include fatigue outcomes have primarily focused on Big Five personality traits or circadian preference and type (De Gucht et al., 2003; Easton et al., 2025; Farzianpour et al., 2015; Iskra-Golec et al., 1995; Karahan et al., 2020; Saksvik-Lehouillier et al., 2012; Saksvik-Lehouillier et al., 2013; West et al., 2007). Among these, only two studies were based on longitudinal designs (Saksvik-Lehouillier et al., 2012; West et al., 2007), and one comprised a randomized control trial considering five nights of consecutive simulated night shift (Easton et al., 2025).

These limitations, the disparate operationalizations of fatigue, and the mixed findings within personality trait research indicate that the roles of adverse childhood experiences and personality traits in relation to excessive fatigue among nurses remain insufficiently explored. Further investigation is thus warranted to clarify relationships between these factors and fatigue outcomes.

One reason for the research gaps discussed above may be due to focus within academic literature on modifiable factors such as schedule characteristics (Peršolja, 2023). While this emphasis is important for intervention development, it may inadvertently obscure the role of non-modifiable individual factors, which may also contribute meaningfully to fatigue risk among nurses. Adverse childhood experiences are likely to be unmodifiable in a work context as they have occurred prior to adult working life. Personality traits are considered relatively stable during adulthood (Burke et al., 2006; Merikanto et al., 2024; Pallesen et al., 2021) and may also be difficult to alter from an occupation health standpoint. Examples of personality traits include the Big Five traits (extroversion, agreeableness, conscientiousness, neuroticism, and intellect/imagination), morningness-eveningness, languidity and flexibility, and some argue workaholism (Burke et al., 2006; Donnellan et al., 2006; Merikanto et al., 2024; Pallesen et al., 2021).

Despite adverse childhood experiences and personality traits being difficult to modify in a workplace setting, their role in fatigue should not be overlooked. Therefore, this study’s objective was to investigate the extent to which Big Five traits, morningness-eveningness, flexibility and languidity, and workaholism associate with excessive fatigue in nurses. We hypothesized that adverse childhood experiences would associate with higher odds of excessive fatigue; that personality traits—particularly neuroticism and languidity—would predict excessive fatigue; and that eveningness would positively associate with excessive fatigue.

## 2 Methods

### 2.1 Study design and population

SUSSH was initiated in 2008/2009, inviting 6,000 randomly selected nurses from the Norwegian Nurses Organization. The sample included nurses who graduated within five different time frames (<12 m, 1–3y, >3–6y, >6–9y and >9–12y). Initial and follow-up questionnaires were sent by post, with 2,059 of 5,400 nurses responding in wave 1 (38.1% response rate). An additional 2,741 recently graduated nurses were invited in 2009, 905 (33.0%) agreed, forming a baseline cohort of 2,964 nurses.

In 2021 (wave 12), SUSSH transitioned to a digital format, with 1,040 participants consenting to continue. Links to follow-up questionnaires are now sent annually via email, excluding those who have died, withdrawn, or have unknown email addresses. Respondents are entered into a lottery for a 500 NOK gift card with 25 winners annually. Research was conducted post approval from the Regional Committee for Medical Research Ethics in Western Norway (No. 088.88). Each participant provided written informed consent. More information is available at www.sussh.no or from past studies (Hiestand et al., 2023; Hiestand et al., 2024; Pallesen et al., 2021). The authors have no competing interests to declare.

**Figure 1:**
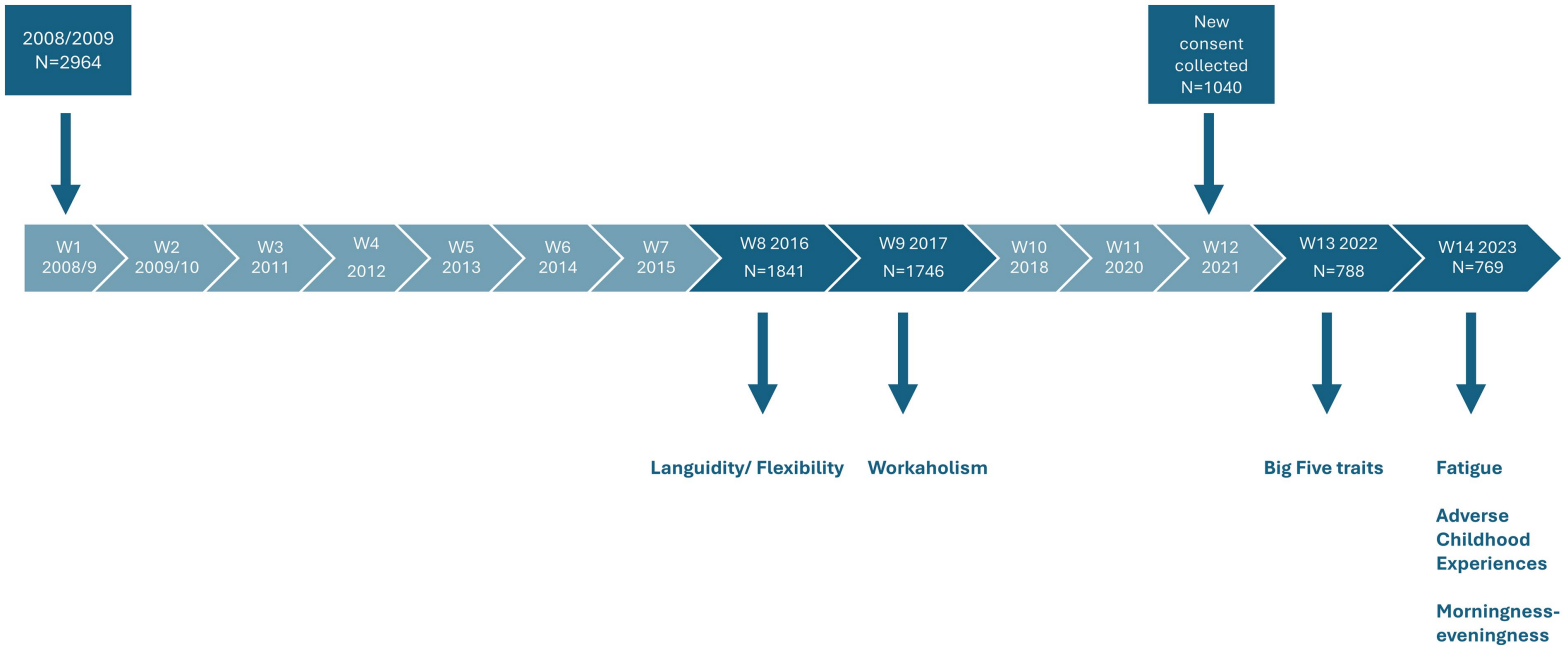
Languidity/flexibility (circadian type) measured with the revised circadian type inventory workaholism measured with the Bergen work addiction scale. Big five traits measured with the mini-international personality item pool (Mini-IPIP). Fatigue measured with the Chalder fatigue questionnaire. Adverse childhood experiences measured with four questions from Haugland et al. (2021). Morningness-eveningness (circadian preference) measured with the Horne and Östberg morningness-eveningness questionnaire-reduced version.

Each wave of SUSSH contains different instruments and questions. The present study utilizes data from waves 8 (2016), 9 (2017), 13 (2022), and 14 (2023; see Figure 1). In 2023 the response rate was 74.0% (*N* = 769), with 748 completing the Chalder Fatigue Questionnaire. Inclusion criteria for the current study were answering the Chalder Fatigue Questionnaire and not being pregnant in 2023. Consequently, the final analytic sample included 741 participants.

### 2.2 Independent variables

#### 2.2.1 Adverse childhood experiences

Those with adverse childhood experiences may have a history of dysfunctional family environments or lack of adult support; they may suffer from bad memories, or perceive their childhood as difficult (Haugland et al., 2021). In line with past research in the Norwegian context (Haugland et al., 2021), adverse childhood experiences were measured using four questions evaluating these areas:

1. Did you experience a lot of arguing, turmoil, conflicts, or difficult communication in your childhood home?
2. Growing up, did you have a trusted adult from whom you could get support?
3. Do you struggle with bad memories from your childhood, due to loss, betrayal, neglect, violence, ill-treatment, or abuse?
4. When you think about your childhood/upbringing, how would you describe it?

Participants responded on a five-point Likert scale from “not at all” to “to a very large degree” on questions 1–3 and from “very good” to “very difficult” for Question 4. All questions were coded trichotomously (no = 0, moderate = 1, high = 2). Detailed coding of each question is available in Supplementary Table 1. Reporting moderate or high levels of each experience was considered having an adverse childhood experience, these were added together and categorized to create a variable accounting for the number of adverse childhood experiences (“0.” “1–2,” “3–4”).

#### 2.2.2 Personality

##### 2.2.2.1 Big five traits

Personality was assessed by the Mini-International Personality Item Pool (Mini-IPIP; Donnellan et al., 2006), a 20-item instrument based on the five-factor model of personality traits. Each trait (extroversion, agreeableness, conscientiousness, neuroticism, intellect/imagination) is measured by four items. Participants rated statements related to each item on a 5-point scale from very inaccurate (1) to very accurate (5). Each composite trait score ranges 4–20. Scores were categorized as “average,” “low,” or “high” based on the mean and standard deviation, following the International Personality Item Pool (2024) website’s guidelines. Cronbach’s alphas for the Big Five personality traits for this study were 0.82 (extroversion), 0.72 (agreeableness), 0.65 (conscientiousness), 0.75 (neuroticism), and 0.73 (intellect/imagination).

##### 2.2.2.2 Circadian preference

Participants completed the 5-item validated reduced version of the Horne and Östberg Morningness-Eveningness Questionnaire (Adan and Almirall, 1991). This assesses the participant’s preferred wake-up time, bedtime, the time-of-day personal efficiency is maximized, the extent of tiredness they experience within a half-hour of waking, and their self-reported circadian type. Answers on the first four items of the questionnaire could score 1–5, on the fifth item it was possible to score 0–6, therefore total scores ranged 4–25. Participants were categorized as morning-type (18–25), intermediate-type (12–17) or evening-type (4–11). Cronbach’s alpha was 0.64.

##### 2.2.2.3 Circadian type

The 11-item revised Circadian Type Inventory (rCTI) was used to measure aspects of circadian rhythm (Di Milia et al., 2005). Participants were asked about their ability to sleep and work at odd times (flexibility/rigidity scale, measured by five items), as well as difficulties overcoming drowsiness and lethargy following reduced sleep duration (languidity/vigor scale, six items). Responses were provided on a 5-point scale ranging from 1 (“almost never”) to 5 (“almost always”). Scores ranged 5–25 (flexibility) or 6–30 (languidity). Participants were also categorized into four sub-groups using percentile scores (within 25th, >25th–50th, >50th–75th, >75th). Higher scores/percentiles indicate a greater degree of the trait. Cronbach’s alphas were 0.84 (flexibility) and 0.71 (languidity).

##### 2.2.2.4 Workaholism

Bergen Work Addiction Scale (7-items), evaluated workaholism based on addiction components (salience, tolerance, mood modification, relapse, withdrawal, conflict and problems). Responses ranged from “never” = 1 to “always” = 5. Scoring “often” or “always” on 4 or more items is the suggested cut-off and was used to categorize workaholism (no/yes; Andreassen et al., 2012). Cronbach’s alpha was 0.86 in the present study.

#### 2.2.3 Dependent variable

The 11-item Chalder Fatigue Questionnaire (Chalder et al., 1993) assessed fatigue within 30 days of answering using a 4-point Likert scale where higher numbers indicate a higher burden of fatigue (range 0–33). Scores for each item were dichotomized (0,0,1,1) so that options such as “less than usual” or “not more than usual” = 0 and “more than usual” or “much more than usual” = 1 (bimodal scoring). Dichotomized scores were summed and scores ≥4 were categorized as excessive fatigue. Dichotomization of fatigue was specified *a priori*. It was performed in order to identify cases of excessive fatigue according to Chalder’s established bimodal scoring guidelines (Jackson, 2014). This approach has been used consistently in prior studies within this cohort (Eldevik et al., 2013; Flo et al., 2014; Hiestand et al., 2023; Hiestand et al., 2024) in order to facilitate comparability of results and interpretation of findings. Cronbach’s alpha for the Chalder Fatigue Questionnaire was 0.92.

#### 2.2.4 Statistics

Data stems from a longitudinal cohort study. Figure 1 illustrates the timepoints at which each variable was measured. Independent variables (except for morningness-eveningness and adverse childhood events) were assessed before the outcome in 2023. For adverse childhood experiences and morningness–eveningness, both predictors and excessive fatigue were assessed in 2023, and analyses for these variables are therefore cross-sectional and do not allow for firm conclusions about temporal order. Relationships between independent variables and excessive fatigue were analyzed using complete-case logistic regression, excluding cases with missing data.

Each variable’s predictive or associative power was evaluated in separate crude analyses. Subsequently, models were adjusted for age (continuous), sex (female/male), marital status (partnered: yes/no), and presence of children at home (yes/no) in 2023. Odds ratios (OR) and 95% confidence intervals (CI) were reported.

Multicollinearity was assessed using the Variance Inflation Factor (VIF), with no multicollinearity detected (all VIFs < 2.0). Outliers were retained in the analysis. All analyses were conducted using SPSS version 29, with a threshold for statistical significance set at *p* < 0.05.

## 3 Results

### 3.1 Demographics and baseline data

The study population consisted of 651 females (88.5%) and 85 males (11.5%). The average age of nurses in 2023 was 45.7(7.9) years, and the majority (78.9%) were married or cohabitating at that time. Over 40% reported excessive fatigue in 2023 (Table 1). Among the nurses, 44.0% worked in a medical hospital/outpatient clinic in 2023. Other work settings reported included 10.0% in psychiatric hospital/outpatient clinic/ambulatory care, 8.0% in nursing homes, 6.1% in home care, 5.5% in child healthcare clinics, and 26.5% in “other” nursing positions.

**Table 1:** Demographics, adverse childhood experiences, personality and fatigue among 741 Norwegian nurses participating in the survey of shift work, sleep and health, wave 14 (2023).

Variables	Percent/mean (SD)
Sex (*n* = 736)	
Women (*n =* 651)	88.5%
Men (*n =* 85)	11.5%
Age (*n* = 740)	45.7 (7.9)
30–39 (*n* = 198)	26.8%
40–49 (*n* = 338)	45.7%
50–59 (*n* = 151)	20.4%
60–75 (*n* = 53)	7.2%
Marital status (*n* = 741)	
Not married/partnered (*n* = 156)	21.1%
Married or partnered (*n* = 585)	78.9%
Children at home (*n* = 741)	
No (*n* = 245)	33.1%
Yes (*n* = 496)	66.9%
Adverse childhood experiences^1^	
Number of adverse childhood experiences (*n* = 741)	
None (*n* = 176)	23.8%
One (*n* = 261)	35.2%
Two (*n* = 144)	19.4%
Three (*n* = 71)	9.6%
Four (*n* = 89)	12.0%
Dysfunctional family environment (*n* = 741)	
Not at all (*n* = 201)	27.1%
To a very small degree (*n* = 200)	27.0%
To a small degree (*n* = 202)	27.3%
To a large degree (*n* = 100)	13.5%
To a very large degree (*n* = 38)	5.1%
Trusted adult (*n* = 741)	
To a very large degree (*n* = 324)	43.7%
To a large degree (*n* = 264)	35.6%
To a small degree (*n* = 86)	11.6%
To a very small degree (*n* = 39)	5.3%
Not at all (*n* = 28)	3.8%
Struggle with bad memories (*n* = 741)	
Not at all (*n* = 457)	61.7%
To a very small degree (*n* = 98)	13.2%
To a small degree (*n* = 123)	16.6%
To a large degree (*n* = 41)	5.5%
To a very large degree (*n* = 22)	3.0%
Perceived childhood (*n* = 741)	
Very good (*n* = 351)	47.4%
Good (*n* = 249)	33.6%
Moderate (*n* = 89)	12.0%
Difficult (*n* = 42)	5.7%
Very difficult (*n* = 10)	1.3%
Personality	
Big Five traits^2^	
Extroversion (*n* = 614)	12.7 (3.6)
Agreeableness (*n* = 614)	16.9 (2.6)
Conscientiousness (*n* = 614)	16.7 (2.6)
Neuroticism (*n* = 614)	11.2 (3.6)
Intellect/Imagination (*n* = 614)	13.1 (3.4)
Circadian preference^3^ (*n* = 741)	
MEQr sum score (*n* = 741)	15.1 (3.5)
Definitely morning types (*n* = 13)	1.8%
Moderately morning types (*n* = 176)	23.8%
Intermediate types (*n* = 434)	58.6%
Moderately evening types (*n* = 105)	14.2%
Definitely evening types (*n* = 13)	1.8%
Flexibility^4^ sum score (*n* = 670)	11.9 (4.4)
Within 25th percentile (*n* = 169)	25.2%
>25^th^ – 50th percentile (*n* = 186)	27.8%
>50^th^ – 75th percentile (*n* = 178)	26.6%
>75^th^ percentile (*n* = 137)	20.4%
Languidity^4^ sum score (*n* = 672)	20.0 (3.8)
Within 25^th^ percentile (*n* = 176)	26.2%
>25^th^ – 50^th^ percentile (*n* = 179)	26.6%
>50^th^ – 75^th^ percentile (*n* = 202)	30.1%
>75^th^ percentile (*n* = 115)	17.1%
Workaholism^5^ (*n* = 684)	12.6 (4.8)
No (*n* = 657)	96.1%
Yes (*n* = 27)	3.9%
Excessive fatigue^6^ (*n* = 741)	
No (*n* = 439)	59.2%
Yes (*n* = 302)	40.8%

1 Adverse childhood experiences measured (2023) using four questions used in the HUNT study, performed in Norway (Haugland et al., 2021).

2 Big Five personality traits assessed in 2022 using the Mini-International Personality Item Pool, range 4–20.

3 Horne and Ostberg Morningness-Eveningness Questionnaire-reduced scale measured in 2023. Higher sum scores refer to higher morningness, range 4–25. Categorized into three categories: morning-type (score = 18–25), neither-type (score = 12–17) and evening-type (score = 4–11).

4 The revised Circadian Type Inventory measured in 2016 with 11-items about daily sleep/work habits and preferences. Higher scores indicate possessing the trait to a higher degree, range 5–25 for flexibility and 6–30 for languidity. Participants were categorized into four sub-groups using quartile scores on the respective scales.

5 Workaholism was measured in 2017 with the Bergen Work Addiction Scale, a 7-item scale, range 7–35. Workaholism = scoring “often” or “always” on ≥4 items.

6 Excessive fatigue measured in 2023 with Chalder Fatigue Questionnaire, dichotomized summed score ≥4 considered excessive fatigue.

Many of our participants reported having experienced adverse events in childhood. More than three-fourths of nurses reported at least one adverse childhood experience, with over one-fifth reporting 3–4 adverse childhood experiences (Table 1). Nearly one in five nurses reported a high degree of dysfunctional family environment, and 7% considered their childhood to be either difficult or very difficult (Table 1).

Generally, among our sample, nurses tended to score high on agreeableness (mean = 16.9, SD = 2.6) and conscientiousness (mean = 16.7, SD = 2.6), and as is true of the general population, (Roenneberg et al., 2007) nearly 60% were intermediate types in terms of circadian preference. While flexibility scores were relatively evenly distributed among quartiles, there were less nurses reporting the highest level of languidity (17.1%), and very few reporting workaholic tendencies (3.9%; Table 1).

### 3.2 Bivariate analyses

Bivariate analyses showed significant differences in nurses reporting excessive fatigue based on differing levels of adverse childhood experiences and personality traits. Notably, nearly 70% of nurses reporting no trusted adult during childhood, or a perception of childhood as difficult reported excessive fatigue in 2023, while nearly three-fourths of those struggling with bad memories reported excessive fatigue. Alternately, those having low neuroticism or low languidity were especially likely to be free from excessive fatigue in 2023 (Table 2).

**Table 2:** Excessive fatigue’s associations with adverse childhood experiences and personality among 741 Norwegian nurses participating in the survey of shift work, sleep and health, wave 14 (2023).

Variables	No excessive fatigue (%)	Excessive fatigue (%)	Chi-square (df)^*^	*p*-value
Sex (*n* = 736)			2.902(1)	0.088
Women (*n* = 651)	57.9%	42.1%		
Men (*n* = 85)	68.2%	31.8%		
Age (*n* = 740)			1.541(3)	0.673
30–39 (*n* = 198)	58.1%	41.9%		
40–49 (*n* = 338)	57.7%	42.3%		
50–59 (*n* = 151)	62.3%	37.7%		
60–75 (*n* = 53)	64.2%	35.8%		
Marital status (*n* = 741)			0.00(1)	0.988
Not married/partnered (*n* = 156)	59.6%	40.4%		
Married or partnered (*n* = 585)	59.1%	40.9%		
Children at home (*n* = 741)			0.723(1)	0.395
No (*n* = 245)	61.6%	38.4%		
Yes (*n* = 496)	58.1%	41.9%		
Adverse childhood experiences^1^			14.339(2)	**<0.001**
Number of adverse childhood experiences (*n* = 741)				
0 (*n* = 176)	63.6%	36.4%		
1–2 (*n* = 405)	62.5%	37.5%		
3–4 (*n* = 160)	46.3%	53.8%		
Dysfunctional family environment (*n* = 741)			11.907 (2)	**0.003**
No (*n* = 201)	63.7%	36.3%		
Moderate (*n* = 402)	61.4%	38.6%		
High (*n* = 138)	46.4%	53.6%		
Trusted adult (*n* = 741)			11.677(2)	**0.003**
High (*n* = 588)	61.7%	38.3%		
Moderate (*n* = 125)	53.6%	46.4%		
No (*n* = 28)	32.1%	67.9%		
Struggle with bad memories (*n* = 741)			32.795(2)	**<0.001**
No (*n* = 457)	61.9%	38.1%		
Moderate (*n* = 221)	63.3%	36.7%		
High (*n* = 63)	25.4%	74.6%		
Perceived childhood as difficult (*n* = 741)			23.746(2)	**<0.001**
No (*n* = 600)	63.0%	37.0%		
Moderate (*n* = 89)	50.6%	49.4%		
Difficult (*n* = 52)	30.8%	69.2%		
Personality				
Big Five traits^2^				
Extroversion (*n* = 614)			7.937(2)	**0.019**
Low (*n* = 162)	56.8%	43.2%		
Average (*n* = 256)	54.7%	45.3%		
High (*n* = 196)	67.3%	32.7%		
Agreeableness (*n* = 614)			7.254(2)	**0.27**
Low (*n* = 162)	57.4%	42.6%		
Average (*n* = 244)	65.6%	34.4%		
High (*n* = 208)	53.4%	46.6%		
Conscientiousness (*n* = 614)			9.831(2)	**0.007**
Low (*n* = 172)	50.0%	50.0%		
Average (*n* = 180)	66.1%	33.9%		
High (*n* = 262)	60.7%	39.3%		
Neuroticism (*n* = 614)			59.586(2)	**<0.001**
Low (*n* = 209)	77.0%	23.0%		
Average (*n* = 242)	58.7%	41.3%		
High (*n* = 163)	37.4%	62.6%		
Intellect/Imagination (*n* = 614)			1.481(2)	**0.477**
Low (*n* = 195)	62.6%	37.4%		
Average (*n* = 209)	58.9%	41.1%		
High (*n* = 210)	56.7%	43.3%		
Circadian preference (*n* = 741)			14.074 (2)	**<0.001**
MEQr^3^ 3 categories (*n* = 736–741)				
Morning types (*n* = 189)	68.8%	31.2%		
Intermediate types (*n* = 434)	58.3%	41.7%		
Evening types (*n* = 118)	47.5%	52.5%		
Flexibility^4^ (*n* = 670)			13.284(3)	**0.004**
Within 25^th^ percentile (*n* = 169)	49.7%	50.3%		
>25^th^ – 50^th^ percentile (*n* = 186)	64.0%	36.0%		
>50^th^ – 75^th^ percentile (*n* = 178)	67.4%	32.6%		
>75^th^ percentile (*n* = 137)	56.9%	43.1%		
Languidity^4^ (*n* = 672)			51.857(3)	**<0.001**
Within 25^th^ percentile (*n* = 176)	73.9%	26.1%		
>25^th^ – 50^th^ percentile (*n* = 179)	68.2%	31.8%		
>50^th^ – 75^th^ percentile (*n* = 202)	52.5%	47.5%		
>75^th^ percentile (*n* = 115)	35.7%	64.3%		
Workaholism^5^ (*n* = 684)			5.673 (1)	**0.017**
No (*n* = 657)	61.8%	38.2%		
Yes (*n* = 27)	37.0%	63.0%		

*Pearson Chi-Square or Yates’ Correction for Continuity (for 2 × 2 tables) and degrees of freedom (df). Excessive fatigue measured in 2023 with Chalder Fatigue Questionnaire, dichotomized summed score ≥4 considered excessive fatigue. Values in bold are statistically significant *p* < 0.05.

1 Adverse childhood experiences measured (2023) using four questions used in the HUNT study, performed in Norway (Haugland et al., 2021).

2 Big Five personality traits assessed in 2022 using the Mini-International Personality Item Pool, range 4–20.

3 Horne and Ostberg Morningness-Eveningness Questionnaire-reduced scale measured in 2023. Higher sum scores refer to higher morningness, range 4–25. Categorized into three categories: morning-type (score = 18–25), neither-type (score = 12–17) and evening-type (score = 4–11).

4 The revised Circadian Type Inventory measured in 2016 with 11-items about daily sleep/work habits and preferences. Higher scores indicate possessing the trait to a higher degree, range 5–25 for flexibility and 6–30 for languidity. Participants were categorized into four sub-groups using quartile scores on the respective scales.

5 Workaholism was measured in 2017 with the Bergen Work Addiction Scale, a 7-item scale, range 7–35. Workaholism = scoring “often” or “always” on ≥4 items.

### 3.3 Logistic regression analyses

#### 3.3.1 Adverse childhood experiences

Adverse childhood experiences showed clear associations with excessive fatigue. Reporting 3–4 adverse childhood experiences roughly doubled the odds of reporting excessive fatigue as compared to having 0 such experiences (aOR = 2.24). In adjusted analyses, there were significant and dose–response relationships between perception of childhood as difficult and lack of trusted adult and excessive fatigue. Higher degrees of adverse experience were correlated with higher odds of reporting excessive fatigue (Table 3). Further, nurses reporting the highest burdens of each adverse childhood experience had higher odds of reporting excessive fatigue in 2023 with adjusted odds ratios ranging from 2.14 (dysfunctional family environment) to 4.94 (struggling with bad memories from childhood).

**Table 3:** Crude and adjusted logistic regression analyses with excessive fatigue as the dependent variable among 741 Norwegian nurses participating in the survey of shift work, sleep and health, wave 14 (2023).

Variables	Crude	Adjusted
	OR (95% CI)^a^	OR (95% CI)^b^
Adverse childhood experiences^1^		
Number of adverse childhood experiences (*n* = 736–741)		
0 (*n* = 175–176)		
1–2 (*n* = 402–405)	1.05 (0.73–1.52)	1.11 (0.77–1.61)
3–4 (*n* = 159–160)	**2.03** (1.31–3.15)	**2.24** (1.44–3.49)
Dysfunctional family environment (*n* = 736–741)		
No (*n* = 200–201)	1.00 (ref)	1.00 (ref)
Moderate (*n* = 399–402)	1.10 (0.78–1.56)	1.15 (0.81–1.64)
High (*n* = 137–138)	**2.03** (1.30–3.15)	**2.14** (1.37–3.35)
Trusted adult (n = 736–741)		
High (*n* = 585–588)	1.00 (ref)	1.00 (ref)
Moderate (*n* = 124–125)	1.40 (0.95–2.06)	**1.51** (1.02–2.25)
No (*n* = 27–28)	**3.41** (1.52–7.66)	**4.68** (1.97–11.11)
Struggle with bad memories (*n* = 736–741)		
No (*n* = 453–457)	1.00 (ref)	1.00 (ref)
Moderate (*n* = 220–221)	0.94 (0.68–1.31)	0.96 (0.69–1.34)
High (*n* = 63)	**4.78** (2.63–8.69)	**4.94** (2.70–9.03)
Perceived childhood as difficult (*n* = 736–741)		
No (*n* = 597–600)	1.00 (ref)	1.00 (ref)
Moderate (*n* = 88–89)	**1.67** (1.06–2.60)	**1.76** (1.12–2.77)
Difficult (*n* = 51–52)	**3.83** (2.08–7.06)	**4.53** (2.40–8.57)
Personality		
Big Five traits^2^		
Extroversion (*n* = 609–614)	**0.96** (0.91–1.00)	**0.95** (0.91–1.00)
Average (*n* = 254–256)	1.00 (ref)	1.00 (ref)
Low (*n* = 161–162)	0.92 (0.62–1.37)	0.92 (0.62–1.38)
High (*n* = 194–196)	**0.59** (0.40–0.86)	**0.59** (0.40–0.87)
Agreeableness (*n* = 609–614)	1.00 (0.94–1.07)	0.99 (0.93–1.06)
Average (*n* = 241–244)	1.00 (ref)	1.00 (ref)
Low (*n* = 162)	1.41 (0.94–2.13)	1.45 (0.96–2.20)
High (*n* = 206–208)	**1.67** (1.14–2.43)	**1.62** (1.10–2.39)
Conscientiousness (*n* = 609–614)	**0.92** (0.87–0.98)	**0.92** (0.86–0.98)
Average (*n* = 178–180)	1.00 (ref)	1.00 (ref)
Low (*n* = 171–172)	**1.95** (1.27–3.00)	**2.02** (1.30–3.12)
High (*n* = 260–262)	1.26 (0.85–1.88)	1.25 (0.84–1.88)
Neuroticism (n = 609–614)	**1.23** (1.16–1.29)	**1.22** (1.16–1.29)
Average (*n* = 241–242)	1.00 (ref)	1.00 (ref)
Low (*n* = 207–209)	**0.42** (0.28–0.64)	**0.44** (0.29–0.66)
Low (*n* = 209)	**0.42** (0.28–0.64)	
High (*n* = 161–163)	**2.37** (1.58–3.57)	**2.37** (1.56–3.59)
Intellect/Imagination (*n* = 609–614)	1.02 (0.98–1.07)	1.04 (0.99–1.09)
Average (*n* = 207–209)	1.00 (ref)	1.00 (ref)
Low (*n* = 195)	0.86 (0.57–1.28)	0.83 (0.55–1.25)
High (*n* = 207–210)	1.09 (0.74–1.61)	1.20 (0.81–1.78)
Circadian Preferences^3^ (*n* = 736–741)	**0.91** (0.88–0.95)	**0.91** (0.87–0.96)
MEQr^3^ 3 categories (*n* = 736–741)		
Intermediate types (*n* = 433–434)	1.00 (ref)	1.00 (ref)
Morning types (*n* = 187–189)	**0.63** (0.44–0.91)	**0.65** (0.45–0.93)
Evening types (*n* = 116–118)	**1.55** (1.03–2.33)	**1.55** (1.02–2.34)
Flexibility^4^ sum score (*n* = 665–670)	0.99 (0.95–1.02)	0.99 (0.95–1.03)
>75^th^ percentile (*n* = 135–137)	1.00 (ref)	1.00 (ref)
Within 25^th^ percentile (*n* = 166–169)	1.34 (0.85–2.11)	1.27 (0.80–2.02)
>25^th^ – 50^th^ percentile (*n* = 186)	0.74 (0.47–1.17)	0.70 (0.44–1.12)
>50^th^ – 75^th^ percentile (*n* = 178)	0.64 (0.40–1.01)	**0.61** (0.38–0.97)
Languidity^4^ sum score (*n* = 668–672)	**1.18** (1.12–1.23)	**1.17** (1.12–1.23)
Within 25^th^ percentile (*n* = 176)	1.00 (ref)	1.00 (ref)
>25^th^ – 50^th^ percentile (*n* = 176–179)	1.32 (0.83–2.09)	1.34 (0.84–2.12)
>50^th^ – 75^th^ percentile (*n* = 201–202)	**2.56** (1.66–3.96)	**2.46** (1.58–3.83)
>75^th^ percentile (*n* = 115)	**5.10** (3.07–8.48)	**5.01** (2.98–8.39)
Workaholism^5^ (*n* = 680–684)		
Workaholism sum score	**1.10** (1.06–1.13)	**1.09** (1.06–1.13)
No workaholism (*n* = 653–657)	1.00 (ref)	1.00 (ref)
Yes workaholism (*n* = 27)	**2.75** (1.24–6.10)	**2.70** (1.21–6.04)

a Separate crude logistic regression analyses for each independent variable.

b Separate logistic regression analyses for each independent variable adjusted for sex, age, and marital status and children at home at baseline.

1 Adverse childhood experiences measured (2023) using four questions used in the HUNT study, performed in Norway (Haugland et al., 2021).

2 Big Five personality traits assessed in 2022 using the Mini-International Personality Item Pool, range 4–20.

3 Horne and Ostberg Morningness-Eveningness Questionnaire-reduced scale measured in 2023. Higher sum scores refer to higher morningness, range 4–25. Categorized into three categories: morning-type (score = 18–25), neither-type (score = 12–17) and evening-type (score = 4–11).

4 The revised Circadian Type Inventory measured in 2016 with 11-items about daily sleep/work habits and preferences. Higher scores indicate possessing the trait to a higher degree, range 5–25 for flexibility and 6–30 for languidity. Participants were categorized into four sub-groups using quartile scores on the respective scales.

5 Workaholism was measured in 2017 with the Bergen Work Addiction Scale, a 7-item scale, range 7–35. Workaholism = scoring “often” or “always” on ≥4 items.

Excessive fatigue measured in 2023 with Chalder Fatigue Questionnaire, dichotomized summed score ≥4 considered excessive fatigue. Values in bold are statistically significant *p* < 0.05.

#### 3.3.2 Personality traits

##### 3.3.2.1 Big-five traits

Continuous variables for extroversion and conscientiousness associated negatively with excessive fatigue, while neuroticism associated positively. Categorically, high conscientiousness was non-significant whereas low conscientiousness had about twice the odds of excessive fatigue. Neuroticism showed a very clear relationship with excessive fatigue, where low neuroticism halved (aOR = 0.44, 95% CI = 0.29–0.66) while high neuroticism doubled the odds of excessive fatigue (aOR = 2.37, 95% CI = 1.56–3.59), reflecting another dose–response relationship (Table 3).

##### 3.3.2.2 Circadian preference

The continuous variable for circadian preference associated negatively, indicating that morningness was associated with reduced odds of excessive fatigue. In the categorical variable, the pattern remains consistent, with nurses categorized as morning types having lower odds of experiencing excessive fatigue and evening types having higher odds compared to intermediate types.

##### 3.3.2.3 Circadian type

Languidity, measured as a continuous variable, was associated with increased odds of excessive fatigue. There was a dose–response relationship between the degree of languidity and the odds of excessive fatigue in the categorical variable, with aORs ranging from 1.34 (25th–50th percentile) to 5.01 (>75th percentile; Table 3).

##### 3.3.2.4 Workaholism

The continuous variable for workaholism was positively associated with excessive fatigue. Nurses categorized as having workaholism had nearly three times the odds of excessive fatigue assessed 6 years later (Table 3).

## 4 Discussion

This study investigated the relationship between potentially non-modifiable factors and excessive fatigue among nurses. Key strengths include a relatively large sample size, the use of validated instruments to assess personality traits, and the measurement of circadian type, workaholism, and Big Five personality traits at earlier time points than the fatigue outcome. Additionally, the cohort’s homogeneity and size help mitigate potential confounding factors related to socioeconomic status, including income and education. To our knowledge, this is the first study to investigate the relationship between adverse childhood experiences and excessive fatigue among nurses.

Clear associations emerged between adverse childhood experiences as well as personality traits and excessive fatigue, supporting the bulk of our hypotheses. The results supported our assumption that adverse childhood experiences would associate with higher odds of excessive fatigue. Our expectation that personality traits—especially neuroticism and languidity—would predict excessive fatigue was in part supported, as nurses who scored high on neuroticism, languidity, or workaholism were more likely to report excessive fatigue, whereas higher extraversion and conscientiousness offered some protection. However, intellect/imagination did not have any significant predictive relationship with excessive fatigue. Finally, our assumption that eveningness would positively associate with excessive fatigue was supported.

Our study contributes to a small body of literature on adverse childhood experiences in nurses (Bouchard and Rainbow, 2021; Clark and Aboueissa, 2021; Gürsoy and Mechmet, 2023; Jung and Oh, 2020; Xu et al., 2025). It corroborates studies showing associations between adverse childhood experiences and negative outcomes (Heim et al., 2006; Petruccelli et al., 2019; Xu et al., 2025), including multiple concurrent adverse childhood experiences increasing risk for adverse outcomes (Hughes et al., 2017; Xu et al., 2025). Possible mechanisms through which adverse childhood experiences could potentially impact excessive fatigue in adulthood include changes in how the body responds to stress (Dempster et al., 2021), impairment of emotional regulation and coping styles (Wang et al., 2021), or correlations with depression, anxiety (Gürsoy and Mechmet, 2023) and poor sleep quality (Jung and Oh, 2020; Vadukapuram et al., 2022).

Previous research on Big Five personality traits relationships with fatigue has shown mixed results, for example studies indicating extroversion and conscientiousness protect against fatigue (De Vries and Van Heck, 2002; Farzianpour et al., 2015), are unrelated (Michielsen et al., 2007; West et al., 2007), or have a delayed fatiguing effect (Leikas and Ilmarinen, 2017). The current study lends credence to the potential protective properties of these traits against excessive fatigue. Neuroticism’s ties to increased fatigue appear more straightforward (Bouchard and Rainbow, 2021; De Gucht et al., 2003; Michielsen et al., 2007; Stephan et al., 2022; Sørengaard et al., 2019; West et al., 2007), and this study’s findings are no exception.

A 2022 meta-analysis on personality and fatigue found that higher extroversion and conscientiousness correlated with better physical/mental health and sleep, more activity, less pain and lower stress reactivity whereas neuroticism correlated with poorer psychological/physical health and sleep, health-risk behavior, more pain, and metabolic syndrome (Stephan et al., 2022). Anxiety, depression, poor sleep and pain (Hiestand et al., 2023; Hiestand et al., 2024) have previously been shown to be associated with excessive fatigue in the SUSSH cohort, supporting the potential of these factors being on the mechanistic pathway to fatigue development.

The clear dose–response relationship between levels of languidity and excessive fatigue demonstrated in this study supported prior research (Saksvik-Lehouillier et al., 2012; West et al., 2007). However, unlike previous studies (Saksvik-Lehouillier et al., 2013), our study did not find a consistent significant protective pattern between flexibility and excessive fatigue. In the adjusted analysis, nurses in the 50th-75th percentile had lower odds of reporting excessive fatigue compared to the most flexible nurses, which may indicate that flexibility to a certain extent is protective, but that too much flexibility may be detrimental. Conversely, this could be an artifact of analysis, especially considering non-significant crude analysis.

Our study supports previous research in nurses (Karahan et al., 2020) and other studies showing associations between eveningness and negative outcomes such as higher rates of depression, mental distress (Merikanto et al., 2024) and burnout (Pelin et al., 2023). These findings may reflect that eveningness is associated with shorter sleep compared to sleep need on weekdays (Merikanto et al., 2024; Roepke and Duffy, 2010). The significant association between workaholism and excessive fatigue, which might reflect elevated levels of stress (Yang et al., 2020), also supports past research (Clark et al., 2021).

### 4.1 Limitations

This cohort included only approximately 11% men, however, this is in line with the sex distribution of Norwegian nurses (Statistics Norway, 2018). As with all cohort studies involving workers, a healthy worker effect could bias results if fatigued nurses stopped working as a nurse, were less likely to respond, or dropped out of the study. The study’s reliance on self-reported data may also introduce bias and the risk of inaccurate recall and the study design limits the ability to infer causality.

Our participants may have been particularly vulnerable to recall errors, as participants reported adverse childhood experiences retrospectively in adulthood, and since a substantial period may have elapsed since childhood. Under ideal research conditions, adverse childhood experiences would have been assessed contemporaneously in childhood, thereby reducing the risk of recall bias and common method bias.

In addition to recall errors due to the passage of time, self-report can be influenced by participant mood, their perceptions of the task, their desire to please, and their opinion or interest in the topic (Gawron, 2016). We cannot rule out that current fatigue, mood, or other unmeasured factors influenced how participants reported their childhood experiences or circadian preference alongside fatigue. Negative affect, for example, may bias participants toward reporting higher levels of distress across domains, hypothetically inflating associations between self-reported factors (Watson and Clark, 1984).

Factors such as depression, anxiety, sleep disorders, and chronic pain may have acted as confounders or mediators in the observed associations. Ideally, these variables would have been controlled for or examined as potential mediators. However, these potentially confounding variables were not measured at a time between the risk factors and the outcomes. Also, likely loss of power due to low sample sizes in some analyses precluded utilizing many control variables within this study and therefore these variables were not incorporated into our analyses.

Causality cannot be inferred regarding adverse childhood experiences or morningness-eveningness purely due to the cross-sectional nature of their analyses. Still, the remaining constructs were assessed at different points in time, which reduces common methods bias (Podsakoff et al., 2003) and provides stronger evidence of causal relationships (Altman and Royston, 2006).

The results from this study should be interpreted with caution. Relatively large sample sizes such as ours carry the risk of identifying statistically significant differences that may be small and not clinically or practically relevant (Sullivan and Feinn, 2012). Further, odds ratios are not the same, nor should they be interpreted as risk ratios. When the outcome is common they can give the impression of a higher risk than actually exists (Ranganathan et al., 2015). Additionally, dichotomization of continuous scales results in information loss and reduced statistical power, however, some measures have established cut-points that support binary classification (Altman and Royston, 2006). While dichotomizing clinical outcomes has drawbacks, it may be considered appropriate when the threshold is specified *a priori* and reflects a clinically meaningful point on the scale, as was the case in the present study (Hancock and Kent, 2016). Finally, while our findings should be interpreted cautiously, many of the variables examined in this study demonstrated practical significance by meeting or exceeding established criteria (Ferguson, 2016).

### 4.2 Implications

While focus on modifiable factors is natural in the context of public health’s drive to develop strategies to modify risk factors and thereby decrease disease risk (Farouk et al., 2025), non-modifiable factors must also be taken into account. Our findings suggest that both life-course experiences and personality traits need consideration when managing fatigue in this workforce.

Several possible mechanistic pathways between adverse childhood experiences and personality traits and excessive fatigue were discussed. These were not specifically tested within this study but rather offer opportunities for future research. The role of sleep, mental health and pain as potential mediators may be explored. Investigations into Organizational interventions such as introducing a trauma-informed care initiative into the workplace (Williamson et al., 2025), which may build resilience by providing tools for personal wellbeing (Center for Health Care Strategies, 2018), should also be considered in relation to nurse fatigue.

## 5 Conclusion

Our study highlights the relationships between excessive fatigue and factors that are less likely to be modifiable within the context of the workplace. However, given that non-modifiable factors may nonetheless influence individuals’ behaviors and lifestyles (Farouk et al., 2025), we believe this study adds to the knowledge base on fatigue in nurses. While the factors investigated here may be difficult for employers or employees to modify, it may still be possible to mitigate their effects via workplace strategies fostering a supportive working environment such as incorporating a trauma-informed response.

## Data Availability

The data analyzed in this study is subject to the following licenses/restrictions: ethical regulations for this project were set by the Regional Committee for Medical Research Ethics in Western Norway (contact: rek-vest@uib.no). Norwegian regulations indicate data containing potentially sensitive and indirectly identifiable information are not allowed to be shared publicly. The data underlying this article will be shared on reasonable request to a SUrvey of Shift work, Sleep and Health project leader or the Bergen Sleep and Chronobiology Network (BeSCN; post@psysp.uib.no). Requests to access these datasets should be directed to www.uib.no/en/rg/sc/120919/survey-shift-work-sleep-and-health-sussh.
