# Supplementary material for: Adverse childhood experiences and personality traits associate with excessive fatigue in Norwegian nurses
Source: Front Psychol. 2026 Mar 10;17:1771618. doi: 10.3389/fpsyg.2026.1771618 (PMC13014621; doi:10.3389/fpsyg.2026.1771618)
Supplement: Supplementary file 1 [file Table_1.docx]

Supplementary Material

# Supplementary Table 1. Coding for Adverse Childhood Experience Questions

| Adverse Childhood Experience^1^ | “not at all” | “to a very small degree” | “to a small degree” | “to a large degree” | “to a very large degree” |
| --- | --- | --- | --- | --- | --- |
| 1. Dysfunctional family environment (Did you experience a lot of arguing, turmoil, conflicts, or difficult communication in your childhood home?) | 0 | 1 | 1 | 2 | 2 |
| 2. Trusted adult (Growing up, did you have a trusted adult from whom you could get support?) | 2 | 1 | 1 | 0 | 0 |
| 3. Struggle with bad childhood memories (Do you struggle with bad memories from your childhood, due to loss, betrayal, neglect, violence, ill-treatment, or abuse?) | 0 | 1 | 1 | 2 | 2 |
|  | “very good” | “good” | “moderate” | “difficult” | “very difficult” |
| 4. Perceived childhood as difficult (When you think about your childhood/upbringing, how would you describe it?) | 0 | 0 | 1 | 2 | 2 |

**Supplementary Table 1.** ^1^Adverse childhood experiences measured (2023) using four questions used in the HUNT study, performed in Norway (Haugland, 2021).

**
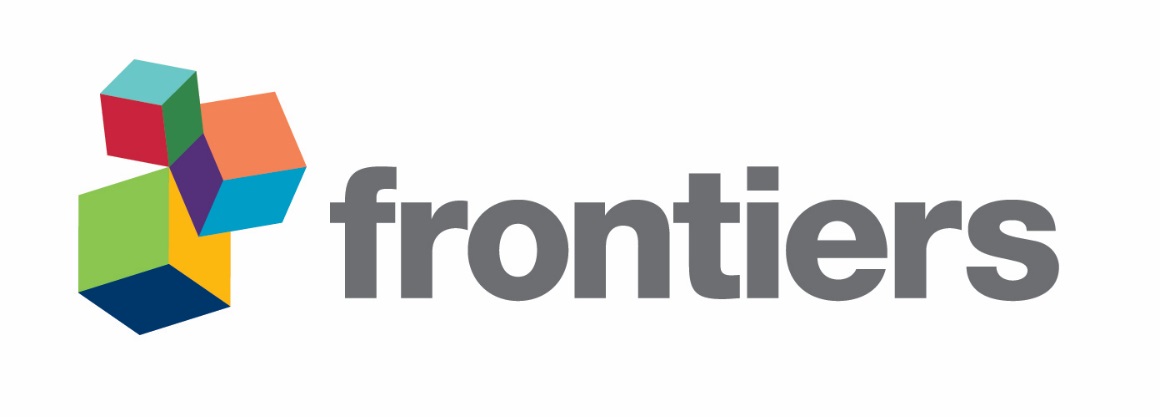
**
